# 

**Published:** 1898-03

**Authors:** 


THE BRISTOL

### Medico-Chirurgical Journal.

A JOURNAL OF THE MEDICAL SCIENCES FOR THE WEST OF ENGLAND AND SOUTH WALES

*PUBLISHED UNDER THE AUSPICES OF*

*THE BRISTOL MEDICO-CHIRURICAL SOCIETY.*

EDITOR:

R. SHINGLETON SMITH, M.D., B.Sc.,

WITH WHOM ARE ASSOCIATED

J. MICHELL CLARKE, M.A., M.D., F. R. CROSS, M.B.,
W. H. HARSANT, AND JAMES SWAIN, M.S., M.D.

ASSISTANT-EDITOR:

L. M. GRIFFITHS.

EDITORIAL SECRETARY:

B. M. H. ROGERS, B.A., M.D., B.Ch.

"Scire est nescire, nisi id me

Scire alius sciret."

*VOL. XVI.*

BRISTOL: J. W. ARROWSMITH.
London: J. & A Churchill, 7 Great Marlborough Street.
1898.

L.H.

R 31
B 8

J.W. Arrowsmith

Publisher

Bristol

### CONTENTS OF VOLUME XVI.

| | Page |
|---|---|
| In Memoriam: AUGUSTIN PRICHARD, F.R.C.S. Eng., M.D. Berlin, L.S.A. ... | 1 |
| The Treatment of Enteric Fever. By J. MICHELL CLARKE, M.A., M.D. Cantab., F.R.C.P. ... | 15 |
| On Some Diseases occuring in Men who Worked in a Sewer. By F. H. EDGEWORTH, M.B., B.A. Cantab., B.Sc. Lond. ... | 33 |
| Isolation after Diphtheria. By BERTRAM M. H. ROGERS, B.A., M.D., B.Ch. Oxon. ... | 101 |
| On Occipital Presentations. By J. G. SWAYNE, M.D. Lond. ... | 108 |
| A Suggestion for the Treatment of Graves's Disease. By W. MACPHUN SEMPLE, M.B., C.M. Glasg. ... | 114 |
| A Case Resembling in Many Respects the Condition known as Mucous Colitis. By E. G. TREVITHICK, M.A., M.D., B.C. Cantab., M.R.C.S. Eng. ... | 120 |
| Thot: An Exalted Surgeon and Physician. By FRANK BUSHNELL, M.D., B.S. Lond., M.R.C.S. Eng. ... | 123 |
| Melancholia. By LIONEL A. WEATHERLY, M.D., C.M. Aberd., M.R.C.S. Eng. ... | 197 |
| Hysterical Paroxysmal Œdema. By F. H. EDGEWORTH, M.B., B.C. Cantab., B.Sc. Lond. ... | 206 |
| Fifty Consecutive Intra-Abdominal Operations. By JAMES SWAIN, M.S., M.D. Lond., F.R.C.S. Eng. ... | 211 |
| A Case of Laparotomy in which a Large Pyosalpinx simulating a Suppurating Tubo-ovarian Cyst was removed. By J. LACY FIRTH, M.S. Lond., F.R.C.S. Eng. ... | 229 |

Page

Progress and Practice. By ROBERT ROXBURGH, M.B. Ed., F.R.C.S. Ed. ... ... ... ... ... ... ... ... ... ... ... ... ... ... 285

Operation Rooms, Past and Present. By Sir WILLIAM MAC CORMAC, Bart., K.C.V.O. ... ... ... ... ... ... .. ... ... ... ... 301

The Radical Cure of Reducible Hernia by Kocher's method of Invagination, with Lateral Displacement of the Neck, of the Sac. By J. LYNN THOMAS, F.R.C.S. Eng. ... ... ... ... ... 307

A Case of Cholecystotomy. By T. CAMPBELL GREY, F.R.C.S. Eng., L.R.C.P. Lond. ... ... ... ... ... ... ... ... ... ... ... 311

A Suggested Method of Copying Sphygmograms. By W. MACPHUN SEMPLE, M.B., C.M. Glas. ... ... ... ... ... ... ... ... ... 312

PROGRESS OF THE MEDICAL SCIENCES ... ... ... 39, 126, 234, 314

REVIEWS OF BOOKS ... ... ... ... ... ... ... 55, 145, 246, 328

NOTES ON PREPARATIONS FOR THE SICK ... .. ... 82, 176, 276, 363

MEETINGS OF SOCIETIES ... ... ... ... ... ... ... 84, 178, 365

THE LIBRARY OF THE BRISTOL MEDICO-CHIRURGICAL SOCIETY ... ... ... ... ... ... ... ... ... 90, 186, 279, 369

LOCAL MEDICAL NOTES ... ... ... ... ... ... ... 94, 193, 282, 378

SCRAPS ... .. ... ... ... ... ... ... ... ... ... 95 195 283, 381

### The Library of the Bristol Medico-Chirurgical Society.

*The following donations have been received since the publication of the list in December:*

*February 28th, 1898.*

| | |
|---|---|
| British Balneological and Climatological Society (1) | 1 volume. |
| T. F. Edgeworth (2) ... ... ... ... ... ... ... ... | 1 ,, |
| Laryngological Society of London (3) ... ... ... ... | 1 ,, |
| Orleans Parish Medical Society (4) ... ... ... | 3 volumes. |
| Medical Society of Pennsylvania (5) ... ... ... | 4 ,, |
| Philadelphia County Medical Society (6) ... ... | 3 ,, |
| Royal Academy of Medicine in Ireland (7) ... ... | 1 volume. |
| Mrs. Greig Smith (8) ... ... ... ... ... ... ... | 46 volumes. |
| R. Shingleton Smith, M.D. (9) ... ... ... ... ... | 7 ,, |
| Trustees of the Smithsonian Institution (10) ... | 7 ,, |
| The Surgeon-General, United States Army (11) | 1 volume. |
| J. Wright and Co. (12) ... ... ... ... ... ... ... | 2 volumes. |

Unbound periodicals have been received from Dr. U. Reber and Dr. R. Shingleton Smith. A framed picture has been presented by Dr. A. E. Aust Lawrence.

### TWENTY-SEVENTH LIST OF BOOKS.

The titles of books mentioned in previous lists are not repeated.

The figures in brackets refer to the figures after the names of the donors, and show by whom the volumes were presented. The books to which no such figures are attached have either been bought from the Library Fund or received through the *Journal*.

| | | |
|---|---|---|
| **Anders, J. M.** | *A Text-Book of the Practice of Medicine.* 2 parts | 1898 |
| **Bashore, H. B.** | *Rural Hygiene* | 1897 |
| **Beard, J.** | *The Span of Gestation and the Cause of Birth* | 1897 |
| **Bice, G. D. Crothers and H. H.** | *Elements of Latin* | 1898 |
| **Browne, O. A.** | *Aneurysms of the Aorta* | 1897 |
| **Bruns, L.** | *Die Geschwülste des Nervensystems* | 1897 |
| *Catalogue (Index) of the Library of the Surgeon-General's Office, United States Army* | (11) 2nd Ser. Vol. II. | 1897 |
| **Clarke, J. J.** | *Surgical Pathology and Principles* | 1897 |
| **Crothers and H. H. Bice, G. D.** | *Elements of Latin* | 1898 |
| **Dakin, W. R.** | *A Handbook of Midwifery* | 1897 |
| **Delagénière, H.** | *Statistique des Opérations pratiquées au Mans du 1er Janvier au 31 Décembre, 1896* | 1897 |
| **Freyberger, L.** | *The Pocket Formulary for the Treatment of Disease in Children* | 1898 |
| **Gasquet, F. A.** | *The Great Pestilence* (A.D. 1348-9) | 1893 |
| **Gordon, H. L.** | *Sir James Young Simpson* (1811-1870) | 1897 |
| **Hall, F. de H.** | *The Medical Examination for Life Assurance* | 1898 |
| **Hutcheson, F.** | *A System of Moral Philosophy.* Vol. I. (8) | 1755 |
| **Lawrie, J. M.** | *Notes on Thirty-two Consecutive Abdominal Sections, with Thirty Recoveries* | 1897 |
| **Lefert, P.** | *Lexique-Formulaire des Nouveautés médicales* | 1898 |
| **Lewers, A. H. N.** | *Diseases of Women* 5th Ed. | 1897 |
| **Macdonald, J. W.** | *Surgical Diagnosis and Treatment* | 1898 |
| **Mitchell, C. P.** | *Dissolution and Evolution and the Science of Medicine* (9) | 1888 |
| **Möbius, P. J.** | *Ueber die Tabes* | 1897 |
| **Morison, A.** | *Cardiac Failure* | 1897 |
| **Niemeyer, [F. v.]** | *Éléments de Pathologie interne et de Thérapeutique.* 2me Éd. (Tr. par L. Culmann et C. Sengel.) 2 vols. in 1 (9) | 1869 |
| **Nicolaysen, L.** | *Studier over Gonorrhoe* | 1898 |
| **Paget, S.** | *Ambroise Paré and His Times* (1510-1590) | 1897 |
| **"Physicist"** | *Human Nature.* Part I. | 1897 |
| **Power, D'A.** | *Some Points in the Anatomy, Pathology, and Surgery of Intussusception* | 1898 |
| " | *William Harvey* | 1897 |
| **Rees, A.** | *Cyclopædia.* 45 vols. (8) | 1819-20 |
| **Scholtz, W. C.** | *The South African Climate* | 1897 |
| *Selected Essays—Syringomyelia, Pemphigus, Yaws, Syphilis, &c.* | | 1897 |
| **Shadwell, A. [Ed.]** | *The Tallerman Treatment* | 1898 |

**Sharp, R. F.** ... *A Dictionary of English Authors* ... ... ... ... ... ... 1897
**Smith, J. G.** ... *Abdominal Surgery.* 6th Ed. (Ed. by J. Swain.) 2 vols. 1897
**Snell, S.** ... ... *The Examination of the Eye* ... ... ... ... ... ... ... 1898
**Sprigge, S. S.** ... *The Life and Times of Thomas Wakley* ... ... ... 1897
*Therapeutics, A System of Practical.* (Ed. by H. A. Hare.) Vol. IV. ... 1897
**Turney, H. G.** ... *Polyneuritis in Relation to Gestation and the Puerperium* 1898
**Waring, H. J.** ... *Manual of Operative Surgery* ... ... ... ... ... ... 1898
**Webster, J. C.** ... *Diseases of Women* ... ... ... ... ... ... ... ... 1898
**Windle, B. C. A.**... *Life in Early Britain* ... ... ... ... ... ... ... 1897
**Wolfenden, R. N.** *Radiography in Marine Zoology* ... ... ... ... ... 1897
**Ziegler, E.** ... ... *A Text-Book of Special Pathological Anatomy.* Sections IX.—XV. (Tr. and Ed. from 8th German Ed. by D. MacAlister and H. W. Cattell) ... ... 1897

---

### TRANSACTIONS, REPORTS, JOURNALS, &c.

American Journal of Obstetrics, The ... ... ... ... ... ...Vol. XXXVI. 1897
American Journal of the Medical Sciences, The... ... ... Vol. CXIV. 1897
American Ophthalmological Society, Transactions of the. Vol. VIII., Part 1. 1897
American Practitioner and News, The ... ... ... Vols. XXIII., XXIV. 1897
American Public Health Association, Reports and Papers of the. Vol. XXII. 1897
American Surgical Association, Transactions of the... ... ...Vol. XV. 1897
Annali di Ostetricia e Ginecologia... ... ... ... ... ... ... ... ... 1897
Archives de Neurologie ... ... ... ... ... 2e Sér., Tomes III., IV. 1897
Archives of Pediatrics, The ... ... ... ... ... ... ... Vol. XIV. 1897
Birmingham Medical Review, The ... ... ... Vols. XLI., XLII. 1897
Boston Medical and Surgical Journal, The ... ... ... Vol. CXXXVI. 1897
Bristol Royal Infirmary Medical Society, The. 2nd Annual Report (2) 1852
British Journal of Dermatology, The ... ... ... ... ... ... Vol. IX. 1897
British Medical Journal, The ... ... ... ... ... ... ... ... Vol. II. for 1897
British Record of Obstetric Medicine and Surgery, The. Vols. I., II. 1848-49
Brooklyn Medical Journal, The ... ... ... ... ... ... ...Vol. XI. 1897
Bulletin de l'Académie royale de Médecine de Belgique—Table générale. 3e Sér. (1867-86) 1897
Centralblatt für Chirurgie ... ... ... ... ... ... ... ... ... ... 1897
Centralblatt für Gynäkologie ... ... ... ... ... ... ... ... ... ... 1897
Centralblatt für innere Medicin ... ... ... ... ... ... ... ... ... 1897
Cincinnati Lancet-Clinic, The ... ... ... ... ... ... Vol. LXXVIII. 1897
Congrès périodique international de Gynécologie et d'Obstétrique. 2me Session. 3 vols. 1897
Directory, Bristol and Clifton... ... ... ... ... ... ... ... ... ... (12) 1898
Directory, Matthews's New History of Bristol or Complete Guide and Bristol. 1794 ... ... ... ... ... ... ... ... ... ... ... ... (12) [1898]
Dublin Journal of Medical Science, The ... ... ... ... ... Vol. CIV. 1897
Écho médical du Nord, L' ... ... ... ... ... ... ... ... ... ... ... 1897
Edinburgh Medical Journal, The ... ... ... ... ... ... N.S., Vol. II. 1897

France médicale, La... ... ... ... ... ... ... ... ... ... ... ... 1897
Gazette des Hôpitaux ... ... ... ... ... ... ... ... ... ... ... 1897
Gazzetta medica Lombarda ... ... ... ... ... ... ... ... ... ... 1897
Giornale internazionale delle Scienze mediche ... ... ... ... ... ... 1897
Hazell's Annual... ... ... ... ... ... ... ... ... ... ... ... 1898
Hospitals—
Johns Hopkins Hospital Reports, The ... ... ... ... Vol. VI. 1897
St. Thomas's Hospital Reports ... ... ... ... N.S., Vol. XXV. 1897
Jahresbericht über die Fortschritte auf dem Gebiete der Chirurgie. II. Jahrgang 1897
Journal of Balneology and Climatology, The ... ... ... (1) Vol. I. 1897
Journal of Cutaneous and Genito-Urinary Diseases ... ... Vol. XV. [1897]
Journal of Laryngology, Rhinology, and Otology, The ... Vol. XII. 1897
Journal of Nervous and Mental Disease, The ... ... ... Vol. XXIV. 1897
Lancet, The... ... ... ... ... ... ... ... ... ... ... Vol. II. for 1897
Laryngological Society of London, Proceedings of the ... (3) Vol. IV. 1897
Laryngoscope, The ... ... ... ... ... ... ... ... ... ... Vol. III. 1897
Mathews' Quarterly Journal of Rectal and Gastro-Intestinal Diseases. Vol. IV. 1897
Medical Age, The ... ... ... ... ... ... ... ... ... Vol. XV. 1897
Medical Directory, The ... ... ... ... ... ... ... ... ... ... ... 1898
Medical Magazine, The ... ... ... ... ... ... ... ... (9) Vol. VI. 1897
Medical News, The ... ... ... ... ... ... ... ... ... Vol. LXXI. 1897
Medical Press and Circular, The ... ... ... ... ... ... [Vol. CXV.] 1897
Medical Record ... ... ... ... ... ... ... ... ... ... Vol. LII. 1897
Medical Society of London, Transactions of the ... ... ...Vol. XX. 1897
Mercy and Truth ... ... ... ... ... ... ... ... ... ... Vol. I. 1897
Münchener medicinische Wochenschrift ... ... ... ... ... ... ... 1897
New Orleans Medical and Surgical Journal. (4) N.S., Vols. XIX., 1892, XXI., 1894. (4) XLIX. 1897
New York Medical Journal, The ... ... ... ... ... ... Vol. LXVI. 1897
Odontological Society of Great Britain, Transactions of the. N.S., Vol. XXIX. 1897
Ophthalmological Society of the United Kingdom, Proceedings of the. Vol. XVII. 1897
Pennsylvania, Transactions of the Medical Society of (5) Vol. XXVII. 1896
Philadelphia County Medical Society, Proceedings of the. (6) Vols. XII., XIII. 1891–92
Post-Graduate, The... ... ... ... ... ... ... ... ... Vol. XII. [1897]
Practitioner, The ... ... ... ... ... ... ... ... ... Vol. LIX. 1897
Progrès médical, Le ... ... ... ... ... ... ... 3e Sér., Tome VI. 1897
Retrospect of Medicine, Braithwaite's... ... ... ... ... Vol. CXVI. 1898
Revue générale d'Ophtalmologie ... ... ... ... ... ... Tome XVI. 1897
Revue hebdomadaire de Laryngologie, d'Otologie et de Rhinologie. Tome XVII., Vol. II. 1897
Royal Academy of Medicine in Ireland, Transactions of the. (7) Vol. XV. 1897
St. Louis Medical and Surgical Journal, The. Vols. LXXII., LXXIII. 1897
Sanitary Record, The ... ... ... ... ... ... ... ... ... ...Vol. XX. 1897
Satellite, The ... ... ... ... ... ... ... ... ... Vols. IV.–VI. 1890–92

Scottish Medical and Surgical Journal, The ... ... ... ... Vol. I. 1897
Smithsonian Report. (10) 1884, 1885, Part 1., 1886, Part 1., 1887, Part 1., 1890-92
Therapeutic Gazette, The ... ... ... ... ... ... ... Vol. XXI. 1897
Travaux pratiques d'Obstétrique et de Gynécologie... ... ... ... ... 1898
West London Medical Journal ... ... ... ... ... ... ... Vol. II. 1897
Whitaker's Almanack ... ... ... ... ... ... ... ... ... ... ... 1898
Wiener klinische Wochenschrift ... ... ... ... ... ... ... ... ... 1897
Year-Book of Treatment, The... ... ... ... ... ... ... ... ... ... 1898

### Local Medical Notes.

### BATH.

Royal United Hospital.—Mr. F. K. Green has been elected an Honorary Surgeon in the place of the late Mr. T. G. Stockwell, and Mr. F. Lace an Honorary Assistant-Surgeon in the place of Mr. Green.

### BRISTOL.

University College.—Mr. J. Paul Bush, M.R.C.S. Eng., has been appointed Lecturer on Operative Surgery in place of Mr. C. F. Pickering, F.R.C.S. Eng., resigned.

The following students have recently passede xaminations:

**University of London.**—*Intermediate Examination* (Second Division)—S. R. Williams. *Excluding Physiology* (Second Division)—P. W. White. *Physiology only* (First Division)—D. T. Price.

**Examining Board in England by the Royal Colleges of Physicians and Surgeons.**—*First Examination. Part III. Elementary Biology*—W. B. Barnes, C. Corfield. *Second Examination. Anatomy and Physiology*—J. C. Norton, J. C. Wadmore. *Final Examination. Surgery*—W. S. V. Stock*; *Medicine*—A. B. Cridland, F. H. Rose,* W. S. V. Stock*; *Midwifery*—E. G. Bunbury, A. B. Cridland, G. Irvine, F. C. Morgan, R. Willcox, W. S. V. Stock.*

**Indian Medical Service.**—W. R. Battye, J. W. Watson.

The Convalescent Home.—This Bristol Memorial of Her Majesty's sixty years' reign is now to all intents and purposes an accomplished fact. After searching for a suitable site or habitation, the Sub-Committee appointed for this object reported that, subject to the approval of the General Committee, they had arranged to purchase the large school occupied by the Rev. J. Wilkinson, with the four and a half acres of ground adjacent, the sum asked for and approved of being £20,000. This magnificent building, which all Bristolians have long known as Waynflete, so named after the founder of Magdalen College, Oxford, is close to Durdham Down, and can, with very little alteration, be made into a suitable home for convalescents.

The total sum required for the endowment of the institution is nearly obtained, and we understand that Mr. E. P. Wills, who it will be remembered offered a house that proved too small and otherwise unsuitable, has given the necessary money for the purchase of the building.

* Completes Examination.

### International Congress of Hygiene and Demography.

Dr. Amalio Gimeno, Professor at the Faculty of Medicine of Madrid, who is the Secretary-General of the Congress, has sent us much interesting information in reference to it. This, the ninth Congress, is to be held this year at Madrid from the 10th to the 17th of April. Besides the important technical work of the Congress, much attention has been devoted to the pleasure of visitors, and we should strongly recommend those of our readers who can go to do so. The Congress is under the patronage of the King and the Queen-Regent, who will entertain the members at a reception. The Government and the Municipality are also organising receptions. Special concessions for getting to Madrid have been granted, and many tempting excursions have been arranged. Membership can be obtained by the payment of £1 to Sir Douglas Galton, 74a Margaret Street, London, who will give any further information that may be required.

### SCRAPS

### Bristol Medico-Chirurgical Society.

### President.

J. E. SHAW, M.B., C.M. Ed.

### President-Elect.

ROBERT ROXBURGH, M.B. Ed.

### Honorary Secretary.

J. PAUL BUSH.

### Committee.

*Past Presidents.*

J. G. SWAYNE, M.D. Lond.
E. LONG FOX, M.D. Oxon.
W. JOHNSTONE FYFFE, M.D., B.A. Dub.
F. POOLE LANSDOWN.
L. M. GRIFFITHS.
R. SHINGLETON SMITH, M.D., B.Sc. Lond.
NELSON C. DOBSON.
S. H. SWAYNE.
F. R. CROSS, M.B. Lond.
E. MARKHAM SKERRITT, M.D., B.S., B.A. Lond.
A. J. HARRISON, M.B. Lond.
ARTHUR W. PRICHARD.
A. E. AUST LAWRENCE, M.D., C.M. Aberd.

J. MICHELL CLARKE, M.D., M.A. Cantab.
JOHN DACRE.
W. H. HARSANT.
B. M. H. ROGERS, M.D., B.Ch., B.A. Oxon.
G. MUNRO SMITH.
H. WALDO, M.D., C.M. Aberd.

Medical Practitioners wishing to join the Society are requested to communicate with the Honorary Secretary, J. PAUL BUSH, Vyvyan House, Clifton Park, Clifton, Bristol. The Annual Subscription is 21/-.

---

*Extracts from Journal By-laws.*

4.—The *Journal* shall be delivered free to Subscribers and also to Members of the Society whose subscriptions are not in arrear.

6.—The Annual Subscription to the *Journal* for those who pay before the 1st of July shall be 5/-, post free, or 6/- if paid after that date.

---

Communications intended for insertion in the *Journal* should be sent to the Editor, Dr. SHINGLETON SMITH, Deepholm, Clifton Park, Clifton, Bristol.

Books for Review and Exchange Journals should be sent to the Assistant-Editor, L. M. GRIFFITHS, 9 Gordon Road, Clifton, Bristol.

Communications referring to the delivery of the *Journal* should be sent to the Editorial Secretary, Dr. BERTRAM ROGERS, 11 York Place, Clifton, Bristol, to whom Subscriptions are to be paid *in advance*.

Cases for Binding Vols. I.–XV. are now ready, and may be obtained, price 7*d*. each (postage 2*d*. extra), upon application to J. W. ARROWSMITH, Quay Street, Bristol; or the *Journal* will be bound, including Case, for 1/3 per volume.

### Bristol Medico-Chirurgical Society.

### PAST PRESIDENTS.

1874—75. FREDERICK BRITTAN, M.D., B.A. Dub.

1875—76. ROBERT WILLIAM COE.

1876—77. SAMUEL MARTYN, M.D. Ed.

1877—78. AUGUSTIN PRICHARD, M.D. Berlin.

1878—79. HENRY EDWARD FRIPP, M.D. Würzburg.

1879—80. WILLIAM MICHELL CLARKE.

1880—81. JOSEPH GRIFFITHS SWAYNE, M.D. Lond.

1881—82. EDWARD LONG FOX, M.D. Oxon.

1882—83. JAMES GEORGE DAVEY, M.D. St. And.

1883—84. WILLIAM JOHNSTONE FYFFE, M.D., B.A. Dub.

1884—85. GEORGE FORSTER BURDER, M.D. Aberd.

1885—86. WILLIAM HENRY SPENCER, M.D., M.A. Cantab.

1886—87. FRANCIS POOLE LANSDOWN.

1887—88. LEMUEL MATTHEWS GRIFFITHS.

1888—89. ROBERT SHINGLETON SMITH, M.D., B.Sc. Lond.

1889—90. NELSON CONGREVE DOBSON.

1890—91. SAMUEL HENRY SWAYNE.

1891—92. FRANCIS RICHARDSON CROSS, M.B. Lond.

1892—93. EDWARD MARKHAM SKERRITT, M.D., B.S., B.A. Lond.

1893—94. JAMES GREIG SMITH, M.B., C.M., M.A. Aberd., F.R.S.E.

1894—95. ALFRED JAMES HARRISON, M.B. Lond.

1895—96. ARTHUR WILLIAM PRICHARD.

1896—97. A. E. AUST LAWRENCE, M.D., C.M. Aberd.

1898.

---

### LIST OF SUBSCRIBERS

TO THE

### Bristol Medico-Chirurgical Journal.

---

*Those marked * are Members of the Society.*

| | |
|---|---|
| Ackland, J. McKno | 24 Southernhay, Exeter. |
| *Ackland, W. R. | 5 Rodney Place, Clifton. |
| Adye, W. J. A. | Church House, Bradford, Wilts. |
| Aldridge, Charles, M.D., C.M. Aberd. | Plympton. |
| Aldous, George F. | Charlton House, Mannamead, Plymouth. |
| Alexander, James, M.D., M.Ch., B.A. Dub. | Bishop's Place, Paignton. |
| Ambrose, J., M.B., C.M. Aberd. | 5 Heath Villas, Eastville, Bristol. |
| *Atchley, Cuthbert | 66 Cotham Road, Bristol. |
| Aveline, H. T. S. | Somerset Asylum, Taunton. |

| | |
|---|---|
| *Ballance, H. Stanley, M.D. Lond. | 4 Royal Terrace, Weston-super-Mare. |
| Bannatyne, Gilbert A., M.D., C.M. Glasg. | 1 Paragon, Bath. |
| *Barclay, W. M. | Queen's Road, Clifton. |
| Baretti, T. G. L'Enardi | 49 Royal York Crescent, Clifton. |
| *Baron, Barclay J., M.B., C.M. Ed. | 16 Whiteladies Road, Clifton. |
| Barrett, W. A. H. | Ottery St. Mary, Devonshire. |
| *Basset, Walter | 24 Stow Hill, Newport, Mon. |
| Batten, Rayner W., M.D. Lond. | 1 Brunswick Square, Gloucester. |
| Batten, W. Smith | Curzon House, Calne. |
| *Beaver, H. Atwood, M.B., B.Ch. Vict. | 6 Downleaze Road, Stoke Bishop. |
| *Beddoe, John, M.D. Ed., B.A. Lond., F.R.S. | The Chantry, Bradford-on-Avon. |
| Benham, Harry A., M.D., C.M. Aberd. | Asylum, Stapleton, Gloucestershire. |
| Bennett, W. S. | Escot, Penzance. |
| *Berry, F. C., M.D., B.Ch., B.A. Dub. | Burnham, Somersetshire. |

### LIST OF SUBSCRIBERS.

| | |
|---|---|
| Berry, James, M.B., B.S. Lond. ... ... ... | 60 Welbeck Street, London, W. |
| Bevan, W. L. P., M.D., C.M. Ed. ... ... ... | Alton, Hampshire. |
| Biamonti, Sebastiano ... ... ... ... ... ... ... | 27 Via Balbi, Genoa, Italy. |
| *Blacker, A. E., M.D. Dub. ... ... ... ... ... | Richmond Hill Avenue, Clifton. |
| *Blagg, Arthur F. ... ... ... ... ... ... ... ... | 6 West Mall, Clifton. |
| Blomfield, Arthur G., M.D. Aberd. ... ... | 34 West Southernhay, Exeter. |
| Board, Edmund C.... ... ... ... ... ... ... ... | 11 Caledonia Place, Clifton. |
| Boyce, James G. ... ... ... ... ... ... ... | The Chipping, Wotton-under-Edge. |
| *Brasher, Charles W. J. ... ... ... ... ... | 128 Cotham Brow, Bristol. |
| Brimacombe, R. William ... ... ... ... ... | Kingswood, Bristol. |
| *Broom, John, M.D. Brux. ... ... ... ... ... | St. Paul's Road, Clifton. |
| Brough, C. A. La T., M.B., B.S., LL.D. Durh. | University College, Bristol. |
| Brown, G. Arthur ... ... ... ... ... ... | Tredegar. |
| Brown, William, M.D., C.M. Glasg. ... ... | Fishponds, Gloucestershire. |
| Browne, J. Walton, M.D., B.A. Royal Univ. I. | 10 College Square North, Belfast. |
| *Bullen, F. St. John ... ... ... ... ... ... | 12 Pembroke Road, Clifton. |
| Burroughs, E. F. H. ... ... ... ... ... ... | 3 Elgin Park, Bristol. |
| *Bush, J. Paul ... ... ... ... ... ... ... ... | Clifton Park, Clifton. |
| *Capron, H. J. ... ... ... ... ... ... ... ... | Princes Road, Clevedon. |
| *Carr, Alexander ... ... ... ... ... ... ... | 150 Wells Road, Bristol. |
| *Carter, T. M. ... ... ... ... ... ... ... ... | 3 Clifton Vale, Clifton. |
| *Carter, W. F. ... ... ... ... ... ... ... ... | 48 Coronation Road, Bristol. |
| *Carwardine, Thomas, M.B., M.S. Lond. ... | 7 Buckingham Place, Clifton. |
| *Cave, Edward J., M.D. Lond. ... ... ... ... | 20 Circus, Bath. |
| Cheves, Alexander B., M.D., M.A. Aberd.... | Millbrook, Devonshire. |
| *Chilton, R. H. ... ... ... ... ... ... ... ... | North Point, Durdham Park, Bristol. |
| Church, Basil... ... ... ... ... ... ... ... ... | Minchinhampton, Gloucestershire. |
| *Clarke, John Michell, M.D., M.A. Cantab. | 28 Pembroke Road, Clifton. |
| Clay, Challoner ... ... ... ... ... ... ... ... | Manor House, Fovant. |
| Clay, R. Hogarth, M.D. Ed. ... ... ... ... | 4 Windsor Villas, Plymouth. |
| Coates, C. J. A. ... ... ... ... ... ... ... ... | Chatteris, Cambridgeshire. |
| Cobbold, C. S. W., M.D. Würzburg... ... ... | Bailbrook House, Bath. |
| Coker, T. V. ... ... ... ... ... ... ... ... ... | 2 Chesterfield Place, Clifton. |
| Cole, Thomas, M.D. Lond.... ... ... ... ... | 18 Circus, Bath. |
| Collins, H. W. ... ... ... ... ... ... ... ... | Wrington. |
| *Cook, Henri, M.D., C.M. Aberd. ... ... ... | 45 Stackpool Road, Bristol. |
| Cooke, A. S. ... ... ... ... ... ... ... ... ... | Badbrook House, Stroud. |
| Corben, C. ... ... ... ... ... ... ... ... ... | The Grove, Caldicot, Monmouthshire. |
| Cory, W. Howard ... ... ... ... ... ... ... ... | 1 The Avenue, Redland. |
| Cossham, W. R., M.D., C.M. Aberd.... ... ... | Cirencester. |
| Cotton, William, M.B., C.M., M.A. Ed. ... | 227 Gloucester Road, Bristol. |
| *Coulter, R. J., M.B. Dub. ... ... ... ... ... | Eye Hospital, Bristol. |
| Cousins, J. Ward, M.D. Lond. ... ... ... ... | Kent Road, Southsea. |
| Cowan, F. S. ... ... ... ... ... ... ... ... ... | 27 Queen Square, Bath. |
| Craddock, Samuel... ... ... ... ... ... ... ... | South Lyncombe, Bath. |
| Crespi, Alfred J. H. ... ... ... ... ... ... | Poole Road, Wimborne. |
| *Cross, F. Richardson, M.B. Lond.... ... ... | Worcester House, Clifton. |
| *Crossman, Edward, M.D. Dur.... ... ... ... | Hambrook, Gloucestershire. |
| *Crossman, F. W. ... ... ... ... ... ... ... ... | Hambrook, Gloucestershire. |
| Crouch, C. Percival, M.B. Lond. ... ... ... | 3 Princes Buildings, Weston-super-Mare. |
| Culross, James, M.B., C.M., M.A. Glasg. ... | 66 Queen Street, Newton Abbot. |
| Cunningham, C. L.... ... ... ... ... ... ... | Chudleigh. |
| *Dacre, John ... ... ... ... ... ... ... ... ... | 14 Eaton Crescent, Clifton. |
| Dalby, Augustus W. ... ... ... ... ... ... ... | Redland House, Frome. |

| | |
|---|---|
| *Davies, D. S., M.D. Lond., D.P.H. Cantab. ... | 60 Oakfield Road, Clifton. |
| Davies, Ebenezer ... | Brunswick House, Swansea. |
| Davies, H. N. ... | Porth, Glamorganshire. |
| Davis, Theodore, M.D. Lond. ... | Beachcroft, Clevedon. |
| Davy, Henry, M.D. Lond. ... | Southernhay House, Exeter. |
| Dening, Edwin ... | Manor House, Stow-on-the-Wold. |
| Devis, Harry F. ... | Wells Road, Bristol. |
| Dickie, James Steel, M.D., C.M. Aberd. ... | Boscombe Park, Bournemouth. |
| *Dobson, Nelson C. ... | 27 Victoria Square, Clifton. |
| Dunbar, Eliza L. Walker, M.D. Zürich ... | 9 Oakfield Road, Clifton. |
| | |
| Eadon, W. F. Bailey ... | Hambrook, Gloucestershire. |
| *Eager, Reginald, M.D. Lond. ... | Northwoods, Winterbourne. |
| Eberle, Emily, M.A., M.B., B.Ch. Dub. ... | 17 Oakfield Road, Clifton. |
| Eddowes, Charles ... | Maddington, Devizes. |
| *Edgeworth, F. H., M.B., B.C., B.A. Cantab., B.Sc. Lond. ... | 35 Pembroke Road, Clifton. |
| Elder, William ... | 7 Trelawney Road, Bristol. |
| *Elliott, Christopher, M.D., B.A. Dub. ... | 88 Pembroke Road, Clifton. |
| Ellis, W. Mc D., M.D. Brux. ... | 9 Bladud Buildings, Bath. |
| Elsworth, R. G., M.B., C.M. Edin. ... | 203 St. Helens Road, Swansea. |
| Essex, J. R. ... | Pontypool, Monmouthshire. |
| Evans, J. Fenton, M.B., C.M. Ed. ... | c/o H. King & Co., 45 Pall Mall, London, S.W. |
| Evans, T. G. C. ... | Budleigh Salterton, Devon. |
| *Ewens, John ... | The Elms, Cotham Hill, Bristol. |
| | |
| Fairbanks, W., M.D., C.M. Ed. ... | Wells, Somersetshire. |
| *Fawcett, E., M.B., C.M. Ed. ... | 141 Redland Road, Bristol. |
| *Fendick, R. George ... | St. Paul's Road, Clifton. |
| Fenwick, Charles ... | Dunsford, Exeter. |
| Finch, Thomas, M.B., B.A. Cantab. ... | Watcombe View, Babbacombe. |
| *Firth, J. L., M.D., B.S. Lond. ... | 20 Richmond Park Road, Clifton. |
| *Fisher, Theodore, M.D. Lond. ... | 25 Pembroke Road, Clifton. |
| *Flemming, C. E. S. ... | Freshford. |
| Ford, Joseph ... | Wedmore. |
| Forty, D. H. ... | Wotton-under-Edge. |
| Fowler, O. H. ... | Cirencester. |
| Fox, Arthur E. W., M.B., C.M. Ed. ... | 16 Gay Street, Bath. |
| *Fox, Bonville B., M.D., M.A. Oxon. ... | Brislington. |
| *Fox, E. Long, M.D. Oxon. ... | Church House, Clifton. |
| Fox, John Alfred ... | Tregea House, Penzance. |
| Fox, T. Colcott, M.B. Lond., B.A. Cantab. ... | 14 Harley Street, London, W. |
| Fraser, Græme B. ... | Beach Road, Weston-super-Mare. |
| *Freeman, J. ... | Cheltenham Road, Bristol. |
| *Fyffe, W. Johnstone, M.D., B.A. Dub. ... | 2 Rodney Place, Clifton. |
| | |
| Gamble, C. H. ... | Barnstaple. |
| *Garland, E. B., M.B., C.M. Edin. ... | 92 Hampton Road, Redland. |
| Garner, John E., M.D., C.M. Aberd. ... | 6 Wenckley Square, Preston, Lancashire. |
| Genge, T. T. ... | 21 Whiteladies Road, Bristol. |
| Gent, W. C. ... | 170 Wells Road, Bristol. |
| *Gerrish, D. G. ... | Royal Infirmary, Bristol. |

### LIST OF SUBSCRIBERS.

| | |
|---|---|
| *Gibbs, Alfred N. Godby | 52 Whiteladies Road, Clifton. |
| Gidley, G. G. | Cullompton, Devon. |
| Goodridge, Henry F. A., M.D. Lond. | 10 Brock Street, Bath. |
| Goss, T. Biddulph | 1 Circus, Bath. |
| Grace, Alfred | Chipping Sodbury. |
| Grace, Edward Mills | Thornbury, Gloucestershire. |
| Grace, W. G. | Ashley Grange, Ashley Down, Bristol. |
| Gratte, C. Brooke | 3 Lansdown Place, Newport, Mon. |
| Grey, T. C. | Church House, St. Neot's. |
| *Griffiths, J. S. | 25 Redland Park, Bristol. |
| *Griffiths, L. M. | 9 Gordon Road, Clifton. |
| Griffiths, P. Rhys, M.B., B.S. Lond. | 71 Newport Road, Cardiff. |
| Groves, J., M.B., B.A. Lond. | Carisbrooke. |
| *Gubbin, W. B. T., M.D., C.M. Edin. | 15 Redland Grove, Bristol. |

| | |
|---|---|
| Hadwen, W. R., M.D. St. And. | 34 Brunswick Square, Gloucester. |
| *Hailes, Clements D. G., M.D., C.M. Ed. | 27 Alma Road, Clifton. |
| *Haines, A. H. | 8 Cotham Road, Bristol. |
| Hale, Edmund T. | The Grange, Chew Magna. |
| Halliwell, T. O. | Blagdon, Somerset. |
| Halpin, W. C. | Parkend, Gloucestershire. |
| Handfield-Jones, Montagu, M.D. Lond. | 35 Cavendish Square, London, W. |
| Hardwick, A., M.B. Dur. | Newquay, Cornwall. |
| Harper, Joseph | Bear Street, Barnstaple. |
| *Harris, H. Elwin, B.A., M.B. Cantab. | 13 Lansdown Place, Clifton. |
| *Harrison, A. J., M.B. Lond. | Guthrie Road, Clifton. |
| Harrison, Charles | Keynsham, Somersetshire. |
| Harsant, J. G., M.D., B.S. Lond. | Exeter Road, Bournemouth. |
| *Harsant, W. H. | Tower House, Clifton. |
| *Harvey, Alfred, M.B. Lond. | Westbury-on-Trym. |
| Harvey, J. | Glenside, Leigh Woods. |
| Hawkins-Ambler, G. A. | 162 Upper Parliament Street, Liverpool. |
| Hawkins, Cæsar F. | North Petherton. |
| Hayman, Alfred G. | 1 Pembroke Road, Clifton. |
| *Hayman, C. A., M.D. St. And. | Kingston Villa, Richmond Hill, Clifton. |
| *Heaven, John C., D.P.H. Lond. | 17 Whiteladies Road, Bristol. |
| Hemming, J. Hughes | Kimbolton, St. Neot's. |
| *Henderson, J. H., B.A. New Zeal., M.B., C.M. Ed. | Children's Hospital, Bristol. |
| *Herapath, C. K. C. | Cotham Park, Bristol. |
| *Hetling, H. E. | 30 Elliston Road, Bristol. |
| Hill, Hedley | 1 Redcliff Hill, Bristol. |
| *Hill, Thomas, M.D. St. And. | 138 Cromwell Road, Bristol. |
| *Hill, Walter J. | Edgcumbe Villa, Clevedon. |
| Hoffman, A. W. W. | 30 The Parade, Leamington. |
| Hollings, Edwin T. | Woolacombe, Morthoe. |
| Horder, T. Garrett | 10 Windsor Terrace, Cardiff. |
| Hospital, Bristol General (*Sec.*, W. Thwaites). | |
| Hospital, Taunton and Somerset (*House-Surgeon*, W. Drake). | |
| Houghton, Leonard F. | East Looe, Cornwall. |
| Howells, William, M.B., C.M. Glasg. | Church House, Talgarth, Breconshire. |
| Howse, William | 8 London Street, Swindon, Wiltshire. |
| Hudson, F. Horace | Beccles, Suffolk. |
| Huggard, W. R., M.D., M.Ch., M.A. Roy. Univ. I. | Davos-Platz, Switzerland. |
| Humphreys, Henry, M.D., M.A. Cantab. | St. Mary Church Road, Torquay. |
| Hunt, A. D. | Millbrook House, Chagford. |
| Hyatt, J. T. | Shepton Mallet. |

| | |
|---|---|
| IMLAY, G. A., M.B., C.M. Aberd. ... ... ... | 1 Cotham Park, Bristol. |
| INSTITUTION, LIVERPOOL MEDICAL (*Resident Librarian*, WILLIAM JONES). | |
| IVES, WILLIAM R. Y. ... ... ... ... ... ... | Portswood Road, Southampton. |
| JACKSON, MARK, M.D. Roy. Univ. I. ... ... ... | Bear Street, Barnstaple. |
| JACOBSON, W. H. A., M.B., M.C., M.A. Oxon. | 66 Great Cumberland Place, London, W. |
| JAMISON, A., M.B., B.Ch., M.A. Roy. Univ. I. ... | 66 Woodstock Road, Belfast. |
| JESSOP, CHARLES MOORE ... ... ... ... ... | Clare Lodge, Redhill. |
| JONES, EVAN ... ... ... ... ... ... ... ... | Ty-mawr, Aberdare. |
| JONES, H. MACNAUGHTON, M.D., M.Ch. Roy.U.I. | 141 Harley Street, London, W. |
| JONES, W. W., M.B., C.M. Ed. ... ... ... ... | 47 Wellington Street, Merthyr Tydvil. |
| *KEMM, F. ST. J.... ... ... ... ... ... ... ... | Worle. |
| KENDALL, BERNARD C. ... ... ... ... ... ... | Cross Street, Helston, Cornwall. |
| *KENDALL, H. W. ... ... ... ... ... ... ... | 9 King's Parade, Clifton. |
| KENNEDY, W. P., M.D., B.Ch., B.A. Dub. ... | High Street, Lydney. |
| *KING, PRESTON, M.D. Cantab. ... ... ... ... | 29 Gay Street, Bath. |
| KINNEIR, R. ... ... ... ... ... ... ... ... | The Tower House, Malmesbury. |
| *LACE, F. ... ... ... ... ... ... ... ... ... | 5 Paragon, Bath. |
| LANCASTER, E. LE CRONIER, B.A., M.B., B.Ch. Oxon. ... ... ... ... ... ... ... ... ... | Winchester House, Swansea. |
| LANGRIDGE, F. W. ... ... ... ... ... ... ... | Croftside, Ilfracombe. |
| *LANSDOWN, F. POOLE ... ... ... ... ... ... | 19 Whiteladies Road, Clifton. |
| *LANSDOWN, R. G. P., M.B., B.S. Dur. ... ... | 39 Oakfield Road, Clifton. |
| LAUWERS, DR. ... ... ... ... ... ... ... ... | Courtrai, Belgium. |
| *LAWRENCE, A. E. AUST, M.D., C.M. Aberd. ... | 19 Richmond Hill, Clifton. |
| LAWRIE, J. MACPHERSON, M.D., C.M. Glasg.... | Greenhill, Weymouth. |
| LEACH, J. COMYNS, M.D. Dur., B.Sc. Lond. ... | Sturminster Newton. |
| *LEES, E. LEONARD, M.D., C.M. Ed. ... ... ... | Redland Road, Bristol. |
| LEIGH, W. W. ... ... ... ... ... ... ... ... | Treharris, Glamorganshire. |
| LIBRARY, COLUMBIA MEDICAL ... ... ... ... | 103 State Street, Chicago, U.S.A. |
| LIDIARD, S. A. ... ... ... ... ... ... ... ... | Kimberley Road, Falmouth. |
| *LIGERTWOOD, WALTER ... ... ... ... ... ... | Altona, Elmdale Road, Clifton. |
| LINDSAY, JAMES A., M.D., M.Ch., M.A. Roy.U.I. | 37 Victoria Place, Belfast. |
| LISTER, LORD, P.R.S. ... ... ... ... ... ... | 12 Park Crescent, London, N.W. |
| *LOWE, T. PAGAN ... ... ... ... ... ... ... | 16 Circus, Bath. |
| LUCAS, J. J. S. ... ... ... ... ... ... ... ... | Highlawn, Totterdown, Bristol. |
| *McCREA, P. W., M.D., B.Ch., B.A., B.A.O., B.A. Dub. ... ... ... ... ... ... ... ... ... | 19 Portland Square, Bristol. |
| MAC DONALD, P. W., M.D., C.M. Aberd.... ... | County Asylum, Dorchester. |
| MAC DOUGALL, HENRY R. ... ... ... ... ... | Dunollie, Oban, N.B. |
| McELFATRICK, JOHN ... ... ... ... ... ... | The Chantry, Mere, Wiltshire. |
| MAC ILWAINE, S. W. ... ... ... ... ... ... ... | South Petherton, Somersetshire. |
| *Mc WATTERS, J. C. ... ... ... ... ... ... ... | Almondsbury. |
| MACKIE, J., M.B., LL.D. Aberd., C.M.G. ... | Alexandria, Egypt. |
| MACKINNON, CHARLES, M.B., C.M., M.A. Glasg. | Dyer Street, Cirencester. |
| MACLEAN, EWEN J., M.D., C.M. Ed.... ... ... | 51 Linden Gardens, London, W. |
| MACLEAN, J. CAMPBELL, M.B., C.M. Ed.... ... | Swindon House, Swindon, Wiltshire. |
| MACREADY, J. ... ... ... ... ... ... ... ... | 51 Queen Ann Street, London, W. |
| MARSH, O. E. BULWER ... ... ... ... ... ... | Parkdale, Newport, Mon. |

| | |
|---|---|
| MARSHALL, ARTHUR L., M.B., B.A. Cantab. ... | Thornleigh, Upper Clapton, London. |
| *MARSHALL, HENRY, M.D. Ed., F.R.S.E. ... ... | 28 Caledonia Place, Clifton. |
| *MARTIN, E. F., M.B. Ed. ... ... ... ... ... ... | Victoria House, Weston-super-Mare. |
| MARTYN, G. J. KING, M.D., B.C., B.A. Cantab. | 12 Gay Street, Bath. |
| MASON, S. B. ... ... ... ... ... ... ... ... | 12 Park Terrace, Pontypool. |
| MASON, WILLIAM ... ... ... ... ... ... ... ... | Sedgemoor Cottage, St. Austell. |
| MATTHEWS, CHARLES E., M.D. Dur. ... ... | 51 Pembroke Road, Clifton. |
| MEREDITH, JOHN, M.D. Ed. ... ... ... ... ... ... | Wellington, Somersetshire. |
| *MILLARD, W. J. KELSON, M.D. St. And. ... ... | 2 Tweeddale Terrace, Tonbridge Wells. |
| MITCHELL, TRAFFORD, M.D. ... ... ... ... | Gorseinon, R.S.O., Glamorganshire. |
| *MOLE, HAROLD F. ... ... ... ... ... ... ... ... | Royal Infirmary, Bristol. |
| MONCKTON, WILLIAM ... ... ... ... ... ... ... | Portishead. |
| MORGAN, ALBERT T., M.D. Brux. ... ... ... | 89 Chesterfield Road, St. Andrew's Park, Bristol. |
| MORGAN, JOHN ... ... ... ... ... ... ... ... ... | Langport, Somersetshire. |
| *MORGAN, T. WHITWORTH S. ... ... ... ... | Pill. |
| *MORTON, C. A. ... ... ... ... ... ... ... ... ... | 14 Vyvyan Terrace, Clifton. |
| *MORTON, W. B., M.D. Lond. ... ... ... ... | Brislington House, Brislington. |
| MUNDEN, CHARLES ... ... ... ... ... ... ... ... | Ilminster. |
| *MYLES, G. T. ... ... ... ... ... ... ... ... | 3 Portland Square, Bristol. |
| *NEALE, A. E., M.B., B.S. Dur. ... ... ... ... | Nevil Road, Bishopston. |
| NELL, RICHARD F. ... ... ... ... ... ... ... ... | 33 Windsor Terrace, Penarth. |
| *NEVILL, W. N., M.D., B.Ch., B.A. Dub. ... ... | Southville, Bristol. |
| *NEWMAN, CHARLES ... ... ... ... ... ... ... ... | 156 Cheltenham Road, Bristol. |
| *NEWNHAM, W. H. C., M.B., M.A. Cantab. ... | Chandos Villas, Clifton. |
| NICHOLSON, T. D., C.M. Ed. ... ... ... ... | 2 Whiteladies Road, Clifton. |
| NORMAN, J. H. ... ... ... ... ... ... ... ... ... | Goodleigh, Winkleigh, Devonshire. |
| O'BRIEN, CORNELIUS ... ... ... ... ... ... ... | Down House, Whitehall, Bristol. |
| ODELL, WILLIAM ... ... ... ... ... ... ... ... | Ferndale, Torquay. |
| *OGILVY, ALEXANDER, M.D., B.Ch., B.A. Dub. | Montrose House, Queen's Road, Clifton. |
| *ORMEROD, H. LAWRENCE, M.B., B.Ch., B.A.O., R.U.I. ... ... ... ... ... ... ... ... ... | Foxstones, Westbury-on-Trym. |
| PAGE, G. SHEPLEY ... ... ... ... ... ... ... ... | 78 Old Market Street, Bristol. |
| *PARKER, GEORGE, M.D., M.A. Cantab. ... ... | 14 Pembroke Road, Clifton. |
| PARRY, J. H. ... ... ... ... ... ... ... ... ... | 199 Cheltenham Road, Bristol. |
| PARSONS, FRANCIS J. C. ... ... ... ... ... ... ... | King Square, Bridgwater. |
| PARSONS, H. F. ... ... ... ... ... ... ... ... ... | The Heath, Stoke Bishop. |
| *PARSONS, J. ST. JOHN G. ... ... ... ... ... ... | 6 Hillside, Cotham. |
| PATERSON, DONALD ROSE, M.D., C.M. Ed. ... | 18 Windsor Place, Cardiff. |
| PEAKE, ARTHUR W. ... ... ... ... ... ... ... ... | 211 Gloucester Road, Bristol. |
| PEAKE, G. ARTHUR ... ... ... ... ... ... ... ... | Rodney Place, Cheltenham. |
| *PENNY, W. J. ... ... ... ... ... ... ... ... ... | Coombe, West Crewkerne, Somerset. |
| *PERRY, W. A. ... ... ... ... ... ... ... ... ... | 13 Dowry Square, Bristol. |
| PHILLIPS, EDWARD PICTON ... ... ... ... ... | High Street, Haverfordwest. |
| PHILLIPS, J. A. ... ... ... ... ... ... ... ... ... | 72 Carlisle Street, Cardiff. |
| PHILLIPS, SUTHERLAND REES, M.D., M.Ch. Roy. Univ. I. ... ... ... ... ... ... ... ... | St. Anne's Heath, Virginia Water, Surrey. |
| *PICKERING, C. F. ... ... ... ... ... ... ... ... | 12 Berkeley Square, Bristol. |
| PLAISTER, W. H. ... ... ... ... ... ... ... ... | High Road, Tottenham. |
| *POCOCK, H. I. ... ... ... ... ... ... ... ... ... | The Barracks, Horfield. |

### LIST OF SUBSCRIBERS.

| | |
|---|---|
| Pollard, Reginald, M.B. Dur. ... ... ... | Southlands, Torquay. |
| *Pountney, W. E., M.D., C.M. Ed. ... ... ... | 33a Whiteladies Road, Bristol. |
| Powell, J. J., M.D. Lond. ... ... ... ... ... | 2 Portland Square, Bristol. |
| Powell, William, M.B. Lond. ... ... ... ... | Hill Garden, Torquay. |
| Price, William, M.B. Lond. ... ... ... ... | 40 Park Place, Cardiff. |
| *Prichard, Arthur W. ... ... ... ... ... ... | 6 Rodney Place, Clifton. |
| *Prichard, J. E., M.B., B.A. Oxon. ... ... ... | 243 Cheltenham Road, Bristol. |
| Pring, A., B.A. Cantab. ... ... ... ... ... | Clyde Road, Redland. |
| *Pringle, J. L. K., M.D. Edin. ... ... ... ... | Bridgwater. |
| *Prowse, Arthur B., M.D. Lond. ... ... ... | 5 Lansdown Place, Clifton. |
| Purdy, J. R., M.D. Aber. ... ... ... ... ... | Lower Hutt, New Zealand. |

| | |
|---|---|
| Randolph, Charles ... ... ... ... ... ... ... | St. Michael's, Milverton, Somersetshire. |
| *Rattray, J. M., M.D., C.M., M.A. Aberd. ... | Frome. |
| Rayner, D. C. ... ... ... ... ... ... ... ... | Oriel Villa, Cotham Road. |
| Redwood, T. Hall, M.D., C.M., M.A. Dur. ... | The Lawn, Rhymney, Monmouthshire. |
| Rendall, John ... ... ... ... ... ... ... | Forestside, Lymington. |
| Riddell, Andrew W. ... ... ... ... ... ... | New Brighton, Cheshire. |
| *Rigg, S. E. ... ... .. ... ... ... ... ... | United Hospitals, Bath. |
| Robathan, G. B. ... ... ... ... ... ... ... | The Grove, Risca. |
| Roberts, Frederick T., M.D., B.Sc. Lond. | 102 Hartley Street, London, W. |
| Roderick, Sydney J., M.B., C.M. Ed. ... ... | Llanelly. |
| Rodgers, John W., M.B., C.M. Ed. ... ... | Redfield, Gloucestershire. |
| *Rogers, B. M. H., M.D., B.Ch., B.A. Oxon. ... | 11 York Place, Clifton. |
| *Rossiter, George F., M.B. Lond. ... ... | Cairo Lodge, Weston-super-Mare. |
| Roué, W. Barrett, M.D., M.S. Dur. ... ... | 2 Buckingham Place, Clifton. |
| Routh, R. Henry F. ... ... ... ... ... ... | Bridgewater. |
| Row, F. Everard ... ... ... ... ... ... ... | 9 Tamar Terrace, Devonport. |
| *Roxburgh, Robert, M.B. Ed. ... ... ... | 1 Victoria Buildings, Weston-super-Mare. |
| *Rudge, C. King ... ... ... ... ... ... ... | 145 Whiteladies Road, Bristol. |
| *Rudge, H. T. ... ... ... ... ... ... ... ... | 5 Colston Parade, Bristol. |
| Ruffer, M. Armand, M.D., B.S., M.B. Oxon. | Cairo, Egypt. |
| Rutherford, H. T. ... ... ... ... ... ... | Park Street, Taunton. |

| | |
|---|---|
| Scott, James, M.B., C.M. Ed. ... ... ... ... | 24 Gaolgate Street, Stafford. |
| *Scott, Richard, J. H. ... ... ... ... ... ... | 28 Circus, Bath. |
| Searle, George C. ... ... ... ... ... ... | Brixham, Devonshire. |
| Searle, Richard Burford... ... ... .. ... | Bexley, Dartmouth. |
| Semple, H. F. ... ... ... ... ... ... ... ... | Budleigh Salterton, Devonshire. |
| Semple, W. R. M., M.B., C.M. Glasg. ... ... | Henford Park, Yeovil, Somersetshire. |
| Seward, W. J., M.B. Lond. ... ... ... ... | Asylum, Colney Hatch, London, N. |
| *Shaw, J. E., M.B., C.M. Ed. ... ... ... ... | 23 Caledonia Place, Clifton. |
| Shaw, Jesse, M.D.... ... ... ... ... ... ... | Fort Beaufort, Cape Colony. |
| Sheen, A., M.D. St. And. ... ... ... ... ... | 23 Newport Road, Cardiff. |
| *Sheppard, E. J. ... ... ... ... ... ... ... | 14 Frederick Place, Clifton. |
| Shortridge, Thomas Wood, M.D. Brussels | Honiton. |
| Simons, C. E. G., M.B., C.M. Aberd. ... ... | The Hollies, Merthyr Tydvil. |
| Skelton, Henry, M.D. St. And. ... ... ... | Downend, Gloucestershire. |
| *Skerritt, E. Markham, M.D., B.S., B.A. Lond. | Richmond Hill, Clifton. |
| Skinner, S., M.B., C.M. Aberd. ... ... ... | Tranent Lawn, Clevedon. |
| *Smith, G. Munro ... ... ... ... ... ... ... | 28 Alma Road, Clifton. |
| *Smith, J. Wesley, M.B., C.M. Aberd. ... ... | Boulevard, Weston-super-Mare. |
| Smith, Rev. Reginald, M.A. Cantab. ... ... | Walpole St. Andrew's, Wisbeach. |
| *Smith, R. Shingleton, M.D., B.Sc. Lond. ... | Clifton Park, Clifton. |
| Smith, W. Alexander, M.B., M.A. Oxon. ... | Newport, Essex. |

| | |
|---|---|
| *Smith, W. A. L., M.A., M.B., B.C. Cantab. | 8 Chamberlain Street, Wells. |
| Smyth, H. J. | New Road, South Molton. |
| Society, Cardiff Medical (*Hon. Sec.*, Dr. D. R. Paterson). | |
| Society, Plymouth Medical (*Hon. Lib.*, J. Elliott Square). | |
| Society, Torquay Medical (*Hon. Sec.*, G. Young Eales). | |
| Solly, Reginald V., M.B., B.S. Lond. | 40 Southernhay, Exeter. |
| Solly, S. E. | Colorado Springs, Colorado, U.S.A. |
| Somer, James | Broad Clyst. |
| Soutar, James Greig, M.B., C.M. Ed. | Barnwood House, Gloucester. |
| *Stack, E. H. E., B.A., M.B., B.C. Cantab. | Royal Infirmary, Bristol. |
| Stamp, W. D. | Ridgeway House, Plympton. |
| Statham, R. Whiteside | Cheddar, Somersetshire. |
| *Steele, Charles, M.D. Dur. | 17 Richmond Hill, Clifton. |
| Steele-Perkins, E. B. | 1 Hill's Buildings, Exeter. |
| *Stephenson, J. B., M.B., B.S. Dur. | 19 Park Row, Bristol. |
| Stevens, W. H. | Zetland Road, Bristol. |
| Stoddart, F. Wallis | 1 Park Street, Bristol. |
| Subscription News Co., The | 77 Clark Street, Chicago, U.S.A. |
| *Swain, James, M.D., M.S. Lond. | 4 Victoria Square, Clifton. |
| *Swayne, J. G., M.D. Lond. | 74 Pembroke Road, Clifton. |
| *Swayne, S. H. | 129 Pembroke Road, Clifton. |
| *Swayne, W. Carless, M.D. Lond. | 8 Leicester Place, Clifton. |
| *Symes, J. O., M.D. Lond. | 11 Richmond Hill, Clifton. |
| Symonds, H. P. | 35 Beaumont Street, Oxford. |
| Symons, John | Buriton House, Penzance. |
| | |
| Taylor, C. Bell, M.D. Edin. | 9 Park Row, Nottingham. |
| *Taylor, E. Claude, M.D. Lond. | Children's Hospital, Bristol. |
| *Taylor, James | 36 Alma Road, Clifton. |
| Temple, G. H., M.D. | Weston-super-Mare. |
| Thomas, A. Garrod, M.D., C.M. Ed. | Clytha Park, Newport, Mon. |
| Thomas, J. Lynn | 28 Charles Street, Cardiff. |
| Thomas, Raglan, M.D. Lond. | 13 West Southernhay, Exeter. |
| Thomas, Rowland | The Cwfr, St. Clears, Carmarthenshire. |
| Thomas, W. H. | Maesteg, Glamorganshire. |
| Thomson, Eustace B., M.D., C.M. Glasg. | Albany Place, Plymouth. |
| *Tivy, W. J. | 8 Lansdown Place, Clifton. |
| Tolputt, Percy T. | 4 Downing Street, Farnham. |
| Tosswill, Louis H., M.B., B.A. Cantab. | 28 West Southernhay, Exeter. |
| Trevithick, Edgar, M.A., M.D. Cantab. | 24 Promenade, Cheltenham. |
| Trotter, Leslie B., M.D., C.M. Ed. | Coleford, Gloucestershire. |
| Tyley, R. P., M.D. St. And. | Wedmore. |
| | |
| Vachell, Herbert R., M.D., C.M. Aberd. | 18 Newport Road, Cardiff. |
| Vereker, R. H. | Eastleigh, Curry Rivel. |
| | |
| Wade, Charles H., M.A. Oxon. | Stanfield, Torquay. |
| *Waldo, Henry, M.D., C.M. Aberd. | 19 Pembroke Road, Clifton. |
| *Walker, Cyril H., M.B., B.A. Cantab. | 3 Leicester Villas, Clifton. |
| Wallace, Rev. Canon, M.A. Oxon. | 3 Harley Place, Clifton. |
| Wallace, J., M.B. | 112 York Road, Bedminster. |
| *Wallace, John | 1 Oriel Terrace, Weston-super-Mare. |
| Walsh, Leslie H. | Royal United Hospital, Bath. |
| Ward, J. L. W. | Victoria Street, Merthyr Tydvil. |
| *Wathen, J. Hancocke | 16 York Place, Clifton. |
| Watters, George T. B., M.D., C.M. Ed. | Stonehouse, Gloucestershire. |

| | |
|---|---|
| WAUGH, ALEXANDER ... ... ... ... ... ... | Midsomer Norton. |
| WEATHERLY, LIONEL A., M.D., C.M. Aberd. | Bailbrook House, Bath. |
| WEBBER, WILLIAM W. ... ... ... ... ... ... | Crewkerne. |
| WEDMORE, C. ERNEST, M.B., M.A. Cantab. ... | Stoke Bishop. |
| *WHICHER, A. H. ... ... ... ... ... ... ... | Midsomer Norton. |
| WHITBY, C. J., B.A., M.D. Cantab. ... ... ... | The Spa, Clifton. |
| WICKHAM, W. ... ... ... ... ... ... ... ... | Hill House, Tetbury. |
| WIGAN, C. A., M.D. Dur. ... ... ... ... ... | Portishead. |
| WIGMORE, J., M.D. Roy. Univ. I. ... ... ... | 20 Green Park, Bath. |
| WILCOX, HENRY, M.B. Aberd. ... ... ... ... | Fleet, Hampshire. |
| *WILDING, JAMES, M.D. Dur. ... ... ... ... | 1 Apsley Villas, Kingsdown Parade, Bristol. |
| WILLCOX, R. LEWIS ... ... ... ... ... ... | Warminster. |
| WILLETT, GEORGE G. D. ... ... ... ... ... | Keynsham, Somersetshire. |
| *WILLIAMS, E. C., M.B., B.C., B.A. Cantab. ... | 26 All Saints' Road, Clifton. |
| WILLIAMS, H. EGERTON ... ... ... ... ... | The Mount, Newport, Mon. |
| WILLIAMS, JOHN, M.D. ... ... ... ... ... ... | 20 Windsor Place, Cardiff. |
| *WILLIAMS, LIONEL H. ... ... ... ... ... ... | Thornbury, Gloucestershire. |
| *WILLIAMS, P. WATSON, M.D. Lond.... ... ... | 1 Victoria Square, Clifton. |
| WILLIAMS, PETER ... ... ... ... ... ... ... | Ferryside, Carmarthenshire. |
| WILLIS, W. M.... ... ... ... ... ... ... ... | 15 Bach Place, London, W. |
| *WINDSOR-AUBREY, H. W. ... ... ... ... ... | Coronation Road, Bristol. |
| WINTERBOTHAM, L. ... ... ... ... ... ... | Bays Hill, Cheltenham. |
| *WINTLE, COLSTON ... ... ... ... ... ... ... | 24 Gloucester Road, Bristol. |
| WOODHEAD, G. SIMS, M.D., C.M. Ed. ... ... | 1 Nightingale Lane, London, S.W. |
| WOODS, FRANK ... ... ... ... ... ... ... | Timsbury. |
| WOOLLCOMBE, WALTER L. ... ... ... ... ... | 16 Lockyer Street, Plymouth. |

*Subscribers are requested to send to the Editorial Secretary any change or alteration of address.*

### Bristol Medico-Chirurgical Journal.

**Terms for Advertisements.**

| | £ | s. | d. |
|---|---|---|---|
| Page ... ... ... ... ... ... ... ... ... ... ... ... ... | 1 | 1 | 0 |
| Half Page ... ... ... ... ... ... ... ... ... ... ... ... | 0 | 12 | 6 |
| Quarter Page ... ... ... ... ... ... ... ... ... ... ... | 0 | 7 | 6 |

Insets (2 or 4 pages), 20/-

Upon orders given for four consecutive insertions, a discount of 25 per cent. will be allowed, if payment is made within one month from date of first insertion. Terms for special positions upon application.

*Orders to be sent to the Editorial Secretary*, B. ROGERS, M.D., *Clifton, Bristol.*

### PUBLICATIONS RECEIVED.

From J. W. Arrowsmith, Bristol:
*The Stethoscope.* Bristol Medical Students' Journal. No. 1. January, 1898.

From J.-B. Baillière et Fils, Paris:
*Lexique-Formulaire des Nouveautés Médicales.* Par Paul Lefert. 1898.

From Baillière, Tindall and Cox, London:
*The Tallerman Treatment.* Edited by Arthur Shadwell, M.A., M.B. 1898.

From John Bale, Sons & Danielsson, Limited, London:
*Notes on Thirty-two Consecutive Abdominal Sections, with Thirty Recoveries, performed within the last seventeen months: with observations.* By James Macpherson Lawrie, M.D. 1897.
*The Earliest Recorded Discovery of Thermal Springs.* By Prosser James, M.D. 1897. [Reprint.]

From Friedr. Bayer & Co., Elberfeld:
*Clinical Excerpts.* November, December, 1897.

From J. F. Bergmann, Wiesbaden:
*Jahresbericht über die Fortschritte auf dem Gebiete der Chirurgie.* II. Jahrgang. 1897.

From California Medical College, San Francisco:
*California Medical Journal.* December, 1897, January, 1898.

From J. & A. Churchill, London:
*Polyneuritis in Relation to Gestation and the Puerperium.* By H. G. Turney, M.A., M.D. 1898.
*The Practice of Midwifery.* By D. Lloyd Roberts, M.D. Fourth Edition. 1896.

From Church Missionary Society, London:
*Mercy and Truth.* January—March, 1898.

From The F. A. Davis Company, Philadelphia:
*Rural Hygiene.* By Harvey B. Bashore, M.D. 1897.
*Elements of Latin.* By Geo. D. Crothers, A.M., M.D., and Hiram H. Bice, A.M. 1898.

From Eduardo Dublan, Mexico:
*Boletin del Consejo superior de Salubridad.* Noviembre, Diciembre, 1897, Enero, 1898.

From Dr. Landon B. Edwards, Richmond, U.S.A.:
*Virginia Medical Semi-Monthly.* January 14—February 11, 1898.

From Feret et Fils, Bordeaux:
*Des Adénoïdites chez les Adultes.* Par le Dr. E. J. Moure. 1898.

From Gustav Fischer, Jena:
*On Certain Problems of Vertebrate Embryology.* By John Beard, D.Sc. 1896.
*The Span of Gestation and the Cause of Birth.* By John Beard, D.Sc. 1897.

From MM. Georg & Co., Geneva:
*Médaille commémorative de la Convention de Genève.* [n.d.] [Reprint.]

From Government Printing Office, Washington:
*Index-Catalogue of the Library of the Surgeon-General's Office, United States Army.* Second Series. Vol. II. B—Bywater. 1897.

From Hampton & Co., London:
*The Dentist.* Vol. I. No. 1. January, 1898.

From Drs. P. Hartenberg & P. Valentin, Paris:
*Revue de Psychologie clinique et thérapeutique.* Janvier, 1898.

From Huntley Bros., Weston-super-Mare:
*Differential Diagnosis and Treatment of Coma.* Arranged by George A. Huntley, M.D. [n.d.]

From Ingram Brothers, London:
*English Illustrated Magazine.* January—March, 1898.

From L'Institut de Bibliographie, Paris:
*Gazette médicale de Paris.* 22, 29 Janvier, 5, 12, 19, 26, Février, 5 Mars, 1898.
*Statistique des Opérations pratiquées au Mans, 1896.* Par H. Delagénière. 1897.

From The Johns Hopkins Press, Baltimore:
*The Johns Hopkins Hospital Reports.* Volume VI. 1897.

From H. K. Lewis, London:
*Diseases of Women.* By Arthur H. N. Lewers, M.D. Fifth Edition. 1897.
*Aneurysms of the Aorta.* By Oswald A. Browne, M.A., M.D. 1897.
*Diseases of the Nervous System.* By Charles E. Beevor, M.D. 1898.

### PUBLICATIONS RECEIVED *(continued)*.

From LONGMANS, GREEN, & Co., London:

*Surgical Pathology and Principles.* By J. JACKSON CLARKE, M.B. 1897.
*The Life and Times of Thomas Wakley.* By S. SQUIRE SPRIGGE, M.B. 1897.
*Exercises in Practical Physiology.* By AUGUSTUS D. WALLER, M.D., F.R.S. Parts I., III. 1897.
*Lectures on Physiology.* First Series. *On Animal Electricity.* By AUGUSTUS D. WALLER, M.D., F.R.S. 1897.

From HORACE MARSHALL & SON, London:

*Around the Western Mediterranean. Travel* Extra. No. 1. By DORA M. JONES. [N.D.]

From "THE MEDICAL WORLD" PUBLISHING CO., London:

*The Medical World.* December 11, 1897, March 5, 1898.

From JOHN P. MORTON & COMPANY, Louisville:

*The Evolution and Perfection of the Aseptic Surgical Technique.* By LEWIS S. MCMURTRY, M.D. 1897. [Reprint.]

From THE NEW YORK POST-GRADUATE HOSPITAL, New York:

*Thirteenth Annual Report of the Directors for the Year ending October 1, 1897.*

From YOUNG J. PENTLAND, Edinburgh:

*Edinburgh Medical Journal.* New Series. Vol. II. 1897.
*Examination of the Eye.* By SIMEON SNELL. 1898.
*Diseases of Women.* By J. C. WEBSTER, B.A., M.D. 1898.
*Manual of Operative Surgery.* By H. J. WARING, M.S., M.B., B.Sc. 1898.

From "PICTORIAL" & "CHRONICLE" OFFICES, Bath:

*The Bath Pictorial.* Sixteenth Edition. [1898.]

From G. P. PUTNAM'S SONS, New York:

*Ambroise Paré and his Times, 1510–1590.* By STEPHEN PAGET. 1897.

From THE REBMAN PUBLISHING CO., LIMITED, London:

*A Text-Book of the Practice of Medicine.* By JAMES M. ANDERS, M.D., Ph.D., LL.D. One volume in two. 1898.
*The Pocket Formulary for the Treatment of Disease in Children.* By LUDWIG FREYBERGER, M.D. 1898.
*Surgical Diagnosis and Treatment.* By J. W. MACDONALD, M.D. 1898.

From J. M. RICHARDS, London:

*Medical Reprints.* December, 1897—February, 1898.
*Some Notes on Gastric Disorders.* [Reprints.]

From THE ROXBURGHE PRESS, LIMITED, London:

*Facts about Monte Carlo.* By A GROUP OF SHAREHOLDERS. [N.D.]

From GEORGE R. RYLEY, Halifax:

*The Position of Gynæcology To-day.* By H. MACNAUGHTON-JONES, M.D., M.A.O., M.Ch. 1898. [Reprint.]

From SMITH, ELDER & Co., London:

*Religio Medici, and other Essays.* By Sir THOMAS BROWNE. Edited by D. LLOYD ROBERTS, M.D. 1898.

From SPOTTISWOODE & Co., London:

*The Outlook.* Vol. I. No. 1. February 5, 1898.

From STEENSKE BOGTRYKKERI, Christiania:

*Studier over Gonokokken og Gonorrhoisk Vulvovaginit hos Born.* Af LYDER NICOLAYSEN. 1898.

From T. FISHER UNWIN, London:

*William Harvey.* By D'ARCY POWER. 1897.
*Sir James Simpson.* By H. LAING GORDON. 1897.

From Dr. CLARENCE A. VEASEY, Philadelphia:

*Primary Sarcoma of the Iris; A Statistical Study, with the Report of an Additional Case, in which the growth was successfully removed by Iridectomy.* By CLARENCE A. VEASEY, A.M., M.D. 1897. [Reprint.]

From WESTERN GAZETTE PUBLISHING COMPANY, Denver:

*Western Medical and Surgical Gazette.* December, 1897.

From JOHN WRIGHT & Co., Bristol:

*The Medical Examination for Life Assurance.* By F. de HAVILLAND HALL, M.D. 1898.
*Doctor and Patient: Hints to Both.* By Dr. ROBERT GERSUNY. Translated by A. S. LEVETUS. 1898.

From K. J. WYSS, Berne:

*Illustrirte Rundschau der medicinisch-chirurgischen Technik.* Jahrgang I. 1 Quartalheft. 15 Februar, 1898.

Bristol Medico-Chirurgical Journal,

MARCH, 1898.

### EXCHANGE-LIST.

### AMERICA.

#### ARGENTINE REPUBLIC.

*Weekly.*

*La Semana Médica.* Buenos Ayres: 475, Cerrito.

#### CANADA.

*Monthly.*

*The Canadian Practitioner.* Toronto: The Practitioner Publishing Company.

*The Montreal Medical Journal.* Montreal: The Gazette Printing Company.

#### MEXICO.

*Twice a month.*

*Gaceta Médica.* Mexico: Imprenta de "El Siglo XIX."

#### PERU.

*Twice a month.*

*La Crónica Médica.* Lima: Imprenta y Librería de San Pedro.

#### UNITED STATES.

*Weekly.*

*Boston Medical and Surgical Journal.* Boston: Damrell & Upham.

*Medical Record.* New York: William Wood & Co.

*The Cincinnati Lancet-Clinic.* Cincinnati: 317 West 7th Street.

*The Journal of the American Medical Association.* Chicago: 61 Market Street (Occidental Buildings).

*The Medical and Surgical Reporter.* Philadelphia: The Butler Publishing Company.

*The Medical News.* New York: Lea Brothers & Co.

*The New York Medical Journal.* New York: D. Appleton & Co.

*Twice a month.*

*Pediatrics.* New York: 254 West 54th Street.

*The American Practitioner and News.* Louisville: John P. Morton and Company.

*The Medical Age.* Detroit: William M. Warren.

*Monthly.*

*Archives of Pediatrics.* New York: E. B. Treat & Co.

*Buffalo Medical Journal.* Buffalo: A. T. Brown Printing House.

*Bulletin of the Johns Hopkins Hospital.* Baltimore: The Johns Hopkins Press.

*International Medical Magazine.* Philadelphia: International Publishing Company.

*Journal of Cutaneous and Genito-Urinary Diseases.* New York: The Physicians' Publishing Company.

*Occidental Medical Times.* Sacramento: D. Johnston & Co.

*The American Gynæcological and Obstetrical Journal.* New York: 1 Madison Avenue.

*The American Journal of Obstetrics and Diseases of Women and Children.* New York: William Wood & Co.

*The American Journal of Ophthalmology.* St. Louis: 3036 Locust Street.

*The American Journal of the Medical Sciences.* Philadelphia: Lea Brothers & Co.

*The Brooklyn Medical Journal.* Brooklyn: 260 Hancock Street.

*The Journal of Nervous and Mental Disease.* New York: 25 West 45th Street.

*The Laryngoscope.* St. Louis: 3702 Olive Street.

*The Post-Graduate.* New York: 2nd Avenue and 20th Street.

*The St. Louis Medical and Surgical Journal.* St. Louis: Commercial Printing Company.

*The Therapeutic Gazette.* Detroit: William M. Warren.

*The Monthly Cyclopædia of Practical Medicine.* Philadelphia: The F. A. Davis Co.

*University Medical Magazine.* Philadelphia: University of Pennsylvania Press.

*Bi-monthly.*

*The Journal of Experimental Medicine.* New York: D. Appleton and Company.

*Quarterly.*

*Mathews' Quarterly Journal of Rectal and Gastro-Intestinal Diseases.* Louisville: John P. Morton & Company.

*The Alienist and Neurologist.* St. Louis: Press of Hughes & Company.

*Yearly.*

*Annual of the Universal Medical Sciences.* Philadelphia: The F. A. Davis Company.

*Proceedings of the Philadelphia County Medical Society.* Philadelphia: A. H. Sickler & Co.

*Reports and Papers of the American Public Health Association.* Concord: Republican Press Association.

*Smithsonian Report.* Washington: Government Printing Office.

*The American Year-Book of Medicine and Surgery.* Philadelphia: W. B. Saunders.

*Transactions of the American Association of Obstetricians and Gynecologists.* Philadelphia: William J. Dornan.

*Transactions of the American Dermatological Association.* New York: Geo. L. Goodman & Co.

*Transactions of the American Gynecological Society.* Philadelphia: William J. Dornan.

*Transactions of the American Laryngological Association.* New York: D. Appleton and Company.

*Transactions of the American Ophthalmological Society.* Hartford: The Case, Lockwood & Brainard Co.

*Transactions of the American Pediatric Society.* New York: A. G. Sherwood & Co.

*Transactions of the American Surgical Association.* Philadelphia: William J. Dornan.

*Transactions of the Association of American Physicians.* Philadelphia: William J. Dornan.

*Transactions of the College of Physicians of Philadelphia.* Philadelphia: William J. Dornan.

*Transactions of the Iowa State Medical Society.* Des Moines: The Kenyon Printing and Mfg. Co.

*Transactions of the Kentucky State Medical Society.* Louisville: John P. Morton & Company.

*Transactions of the Medical Society of Pennsylvania.* Philadelphia: The Edwards & Docker Co.

*Transactions of the Medical Society of the State of New York.* Philadelphia: William J. Dornan.

*Transactions of the Michigan State Medical Society.* Grand Rapids: Seymour & Muir Printing Co.

*Transactions of the New Hampshire Medical Society.* Concord: Ira C. Evans.

*Transactions of the New York Academy of Medicine.* Concord: Republican Press Association.

*Transactions of the South Carolina Medical Association.* Charleston: Walker, Evans & Cogswell Co.

### EXCHANGE-LIST (*continued*).

### ASIA.

### CHINA.

*Quarterly.*

*The China Medical Missionary Journal.* Shanghai: The American Presbyterian Mission Press.

### INDIA.

*Monthly.*

*Indian Medical Gazette.* Calcutta: Thacker, Spink & Co.

### JAPAN.

*Monthly.*

*The Sei-I-Kwai Medical Journal.* Tokio: 6 Shin Sakana Chō, Kyōbashi Ku.

### AUSTRALASIA.

### AUSTRALIA.

*Monthly.*

*Intercolonial Medical Journal of Australasia.* Melbourne: Stillwell and Co.

*The Australasian Medical Gazette.* Sydney: 121 Bathurst Street.

### EUROPE.

### AUSTRIA.

*Weekly.*

*Wiener klinische Wochenschrift.* Vienna: Wilhelm Braumüller.

### BELGIUM.

*Twice a month.*

*Journal de Neurologie & d'Hypnologie.* Brussels: A. Gossé & Ce.

*Monthly.*

*Bulletin de l'Académie royale de Médecine de Belgique.* Brussels: F. Hayez.

### BRITISH ISLES.

*Weekly.*

*British Medical Journal.* London: 429 Strand.

*Medical Press and Circular.* London: A. A. Tindall.

*The Clinical Journal.* London: The Medical Publishing Company, Limited.

*The Hospital.* London: The Scientific Press (Limited).

*The Lancet.* London: 423 Strand.

*The Sanitary Record.* London: The Sanitary Publishing Co., Ltd.

*Twice a month.*

*Treatment.* London: The Rebman Publishing Company, Limited.

*Monthly.*

*Edinburgh Medical Journal.* Edinburgh: Young J. Pentland.

*The Birmingham Medical Review.* Birmingham: Hall & English.

*The British Journal of Dermatology.* London: H. K. Lewis.

*The Dublin Journal of Medical Science.* Dublin: Fannin & Co., Ltd.

*The Journal of Laryngology, Rhinology, and Otology.* London: The Rebman Publishing Company, Limited.

*The Medical Chronicle.* Manchester: John Heywood.

*The Scottish Medical and Surgical Journal.* Edinburgh: William F. Clay.

*Transactions of the Odontological Society of Great Britain.* London: John Bale, Sons & Danielsson, Ltd.

*The Practitioner.* London: Cassell & Company, Limited.

*Quarterly.*

*Archives of Surgery.* London: West, Newman & Co.

*The British Gynæcological Journal.* London: John Bale, Sons & Danielsson, Ltd.

*The Northumberland and Durham Medical Journal.* Newcastle-upon-Tyne: The "Daily Journal" Office.

*The Quarterly Medical Journal.* Sheffield: Pawson and Brailsford.

*The West London Medical Journal.* London: John Bale, Sons & Danielsson, Ltd.

*Half-yearly.*

*The Liverpool Medico-Chirurgical Journal.* Liverpool: Medical Institution.

*The Retrospect of Medicine.* London: Simpkin & Co. Limited.

*Yearly.*

*Clinical Society's Transactions.* London: Longmans, Green, and Co.

*Guy's Hospital Reports.* London: J. & A. Churchill.

*King's College Hospital Reports.* London: Adlard & Son.

*Medico-Chirurgical Transactions.* London: Longmans, Green, and Co.

*Obstetrical Transactions.* London: Longmans, Green, and Co.

*Ophthalmological Transactions.* London: J. & A. Churchill.

*St. Bartholomew's Hospital Reports.* London: Smith, Elder & Co.

*St. Thomas's Hospital Reports.* London: J. & A. Churchill.

*The Medical Annual.* Bristol: John Wright & Co.

*The Medical Society's Transactions.* London: Harrison & Sons.

*The Transactions of the Edinburgh Obstetrical Society.* Edinburgh: Oliver and Boyd.

*The Westminster Hospital Reports.* London: J. & A. Churchill.

*The Year-Book of Treatment.* London: Cassell & Company, Limited.

*Transactions of the Dermatological Society of Great Britain and Ireland.* London: H. K. Lewis.

*Transactions of the Epidemiological Society of London.* London: Shaw and Sons.

*Transactions of the Royal Academy of Medicine in Ireland.* Dublin: Fannin & Co., Ltd.

*Year-Book of Scientific and Learned Societies.* London: C. Griffin & Co., Ltd.

*Occasionally.*

*The International Directory of Second-hand Booksellers.* Rochdale: James Clegg.

*Transactions of the British Institute of Preventive Medicine.* London: Macmillan & Company, Limited.

DENMARK.

*Weekly.*

*Hospitalstidende.* Copenhagen: Jacob Lund.

FRANCE.

*Three times a week.*

*Gazette des Hôpitaux.* Paris: 4 Rue de l'Odéon.

*Weekly.*

*Bulletin de l'Académie de Médecine.* Paris: Masson et Cie.

*Gazette hebdomadaire des Sciences médicales de Bordeaux.* Bordeaux: P. Cassignol.

*Gazette des Hôpitaux de Toulouse.* Toulouse: 3 Avenue Lafayette.

*Journal d'Hygiène.* Paris: 30 Rue de Dragon.

*La France médicale.* Paris: 9 Rue des Pyramides.

*La Revue médicale.* Paris: 69 Boulevard Saint-Michel.

*L'Echo médical du Nord.* Lille: Le Bigot Frères.

*Le Progrès médical.* Paris: 14 Rue des Carmes.

*Revue hebdomadaire de Laryngologie, d'Otologie et de Rhinologie.* Paris: Octave Doin.

*Fortnightly.*

*Le Nord médical.* Lille: 209 Rue Léon-Gambetta.

*Twice a month.*

*Gazette de Gynécologie.* Paris: 12 Rue de Châteaudun.

*La Revue médicale.* Paris: F. Sallé.

*Revue de Thérapeutique médico-chirurgicale.* Paris: Félix Alcan.

*Monthly.*

*Archives cliniques de Bordeaux.* Bordeaux: 8 Rue de Cheverus.

*Archives de Neurologie.* Paris: 14 Rue des Carmes.

*Archives provinciales de Chirurgie.* Paris: Institut international de Bibliographie scientifique.

*Revue générale d'Ophtalmologie.* Paris: Masson et Cie.

*Revue médico-chirurgicale des Maladies des Femmes.* Paris: 45 Boulevard Malesherbes.

*Revue pratique d'Obstétrique et de Gynécologie.* Paris: 47 Boulevard Haussmann.

*Quarterly.*

*La Bibliographie scientifique.* Paris: Institut international de Bibliographie scientifique.

GERMANY.

*Weekly.*

*Centralblatt für Chirurgie.* Leipzig: Breitkopf & Härtel.

*Centralblatt für Gynäkologie.* Leipzig: Breitkopf & Härtel.

*Centralblatt für innere Medicin.* Leipzig: Breitkopf & Härtel.

*Münchener medicinische Wochenschrift.* Munich: J. F. Lehmann.

*Monthly*

*Zeitschrift für Krankenpflege.* Berlin: H. Kornfeld.

HOLLAND.

*Weekly.*

*Nederlandsch Tijdschrift voor Geneeskunde.* Amsterdam: F. van Rossen.

ITALY.

*Weekly.*

*Gazzetta Medica Lombarda.* Milan: Fratelli Bocca.

*Twice a month.*

*Giornale Internazionale delle Scienze Mediche.* Naples: Detken & Rocholl.

*Il Policlinico.* Rome: Dante Alighieri.

*Monthly.*

*Annali di Ostetricia e Ginecologia.* Milan: L. F. Cogliati.

*Bi-monthly.*

*Archivio di Ortopedia.* Milan: Pietro Agnelli.

NORWAY.

*Monthly.*

*Norsk Magazin for Lægevidenskaben.* Christiania: Steenske Bogtrykkeri.

PORTUGAL.

*Weekly.*

*A Medicina Contemporanea.* Lisbon: José Antonio Rodrigues.

RUSSIA.

*Weekly.*

*Medycyna.* Warsaw: 38 Rue de Varenne.

*St. Petersburger medicinische Wochenschrift.* St. Petersburg: Carl Ricker.

*Vrach.* St. Petersburg: C. Ricker.

*Monthly.*

*Finska Läkaresällskapets Handlingar.* Helsingfors: Helsingfors Central-Tryckeri.

SPAIN.

*Weekly.*

*El Siglo Médico.* Madrid: Magdalena, 36.

SWEDEN.

*Bi-monthly.*

*Nordiskt Medicinskt Arkiv.* Stockholm: P. A. Norstedt & Söner.

